# Comparison of the safety and immunogenicity of the BNT-162b2 vaccine and the ChAdOx1 vaccine for solid organ transplant recipients: a prospective study

**DOI:** 10.1186/s12879-022-07764-x

**Published:** 2022-10-13

**Authors:** Aziza A. Ajlan, Tariq Ali, Hassan Aleid, Khalid Almeshari, Edward DeVol, Morad Ahmed Alkaff, Layal Fajji, Ali Alali, Dani Halabi, Sahar Althuwaidi, Saad Alghamdi, Asad Ullah, Abdulrahman Alrajhi, Khalid Bzeizi, Reem Almaghrabi, Kris Ann Hervera Marquez, Bilal Elmikkaoui, Eid Albogumi, Haifa Aldakhil, Moheeb Al-Awwami, Dieter C. Broering

**Affiliations:** 1 Clinical Pharmacy Specialist-Solid Organ Transplant. Transplant Clinical Pharmacy Section. Organ Transplant Centre of Excellence, King Faisal Specialist Hospital and Research Centre (KFSHRC), P.O. Box 3354, Riyadh, 11211 Kingdom of Saudi Arabia; 2 Adult Transplant Nephrology, Kidney and Pancreas Health Centre, Organ Transplant Center of Excellence, King Faisal Specialist Hospital and Research Centre, Riyadh, Kingdom of Saudi Arabia; 3 Biostatistics, Epidemiology and Scientific Computing, King Faisal Specialist Hospital & Research Centre, Riyadh, Kingdom of Saudi Arabia; 4 Immunology and Serology Laboratory, King Faisal Specialist Hospital and Research Centre, Riyadh, Kingdom of Saudi Arabia; 5 Clinical Transplant, Organ Transplant Center of Excellence, King Faisal Specialist Hospital and Research Centre, Riyadh, Kingdom of Saudi Arabia; 6 Transplant Coordination Team Leader, Organ Transplant Center of Excellence, King Faisal Specialist Hospital and Research Centre, Riyadh, Kingdom of Saudi Arabia; 7 Transplant Clinical Specialist, Organ Transplant Center of Excellence, King Faisal Specialist Hospital and Research Centre, Riyadh, Kingdom of Saudi Arabia; 8 Medical Microbiology, Microbiology, Pathology and Laboratory Medicine Department., King Faisal Specialist Hospital and Research Centre, Riyadh, Kingdom of Saudi Arabia; 9 Adult Transplant Hepatology, Adult Transplant Hepatology, Organ Transplant Center of Excellence, King Faisal Specialist Hospital and Research Centre, Riyadh, Kingdom of Saudi Arabia; 10 Infectious Diseases, Medicine Department-Riyadh, King Faisal Specialist Hospital and Research Centre, Riyadh, Kingdom of Saudi Arabia; 11 Infectious Diseases, Organ Transplant Centre of Excellence Department-Riyadh, King Faisal Specialist Hospital and Research Centre (KFSHRC), Riyadh, Kingdom of Saudi Arabia; 12 Analytics Data Centre, Organ Transplant Centre of Excellence Department-Riyadh, King Faisal Specialist Hospital and Research Centre, Riyadh, Kingdom of Saudi Arabia; 13 Clinical Analyst, Data Management, Organ Transplant Centre of Excellence Department-Riyadh, King Faisal Specialist Hospital and Research Centre (KFSHRC), Riyadh, Kingdom of Saudi Arabia; 14 Histocompatibility and Immunogenetics Laboratory, Kidney and Pancreas Health Centre Department, Organ Transplant Center of Excellence, King Faisal Specialist Hospital and Research Centre, Riyadh, Kingdom of Saudi Arabia; 15 Organ Transplant Centre, Organ Transplant Center of Excellence, King Faisal Specialist Hospital and Research Centre, Riyadh, Kingdom of Saudi Arabia; 16 College of Medicine, AlFaisal University, Riyadh, Saudi Arabia

**Keywords:** COVID-19, Liver transplant, Kidney transplant, Infection, Immunity vaccine

## Abstract

Severe acute respiratory syndrome coronavirus 2 (SARS-CoV-2) infection and its resulting disease, coronavirus disease 2019 (COVID-19), has spread to millions of people worldwide. Preliminary data from organ transplant recipients have shown reduced seroconversion rates after the administration of different SARS-CoV-2 vaccination platforms. However, it is unknown whether different vaccination platforms provide different levels of protection against SARS-CoV-2. To answer this question, we prospectively studied 431 kidney and liver transplant recipients (kidney: n = 230; liver: n = 201) who received either the ChAdOx1 vaccine (n = 148) or the BNT-162b2 vaccine (n = 283) and underwent an assessment of immunoglobulin M/immunoglobulin G spike antibody levels. The primary objective of the study is to directly compare the efficacy of two different vaccine platforms in solid organ transplant recipients by measuring of immunoglobulin G (IgG) antibodies against the RBD of the spike protein (anti-RBD) two weeks after first and second doses. Our secondary endpoints were solicited specific local or systemic adverse events within 7 days after the receipt of each dose of the vaccine. There was no difference in the primary outcome between the two vaccine platforms in patients who received two vaccine doses. Unresponsiveness was mainly linked to diabetes. The rate of response after the first dose among younger older patients was significantly larger; however, after the second dose this difference did not persist (p = 0.079). Side effects were similar to those that were observed during the pivotal trials.

**Supplementary Information:**

The online version contains supplementary material available at 10.1186/s12879-022-07764-x.

## Introduction

Since the emergence of the coronavirus disease 2019 (COVID-19) pandemic, several vaccine platforms have evolved and emergency use authorization has been filed for their use. Key platforms of these vaccines include mRNA and adenovirus vectors. Adenoviruses, retroviruses, and vaccinia viruses are typically used as carrier vehicles in viral vector vaccines [1].

Transplant recipients remain vulnerable to the development of severe COVID-19, with higher reported morbidity and mortality than the general population [2]. Solid organ transplant recipients and immunosuppressed individuals were excluded from phase 3 trials of all COVID-19 vaccines [3–8]. Studies have looked at the response of mRNA vaccines across solid organ transplant recipients, and showed diminished response. Which has led to recommending a third dose of the vaccine [9, 10]. Furthermore, the immune responsiveness across platforms may vary. No studies have explored the impact of different vaccine platforms on the generated immunity, especially in immunocompromised hosts. The primary objective of the study is to directly compare the efficacy and safety of two different vaccine platforms (i.e., BNT-162b2 vaccine versus ChAdOx1) in solid organ transplant recipients by measuring of immunoglobulin G (IgG) antibodies against the RBD of the spike protein (anti-RBD) two weeks after first and second doses. During this prospective study, we compared the immunogenicity of the two COVID-19 vaccine platforms prospectively.

## Materials and methodS

### Patient population and study design

Patients followed-up at the King Faisal Specialist Hospital and Research Centre who received two doses of either the BNT-162b2 vaccine or the ChAdOx1 vaccine were included in this study. Informed consent was obtained from all participants, and blood samples were obtained according to the follow-up schedule (Additional file 1: Appendix A).

The institutional ethics committee approved this study (RAC# 2211022). The key exclusion criterion for patients was known COVID-19 infection, multi-organ transplant and age < 18 years, receipt of the vaccine before transplant and history of rejection within 6 months preceding vaccine administration.

### Antibody responses

The primary outcome was the measurement of immunoglobulin G (IgG) antibodies against the RBD of the spike protein (anti-RBD) two weeks after first and second doses (Additional file 1: Appendix A). The two-week time point was selected based on previous studies that indicated that antibody titers are expected to peak at those time points [11–13]. The anti-RBD was measured by semi-quantitative anti-spike serologic testing using the Roche Elecsys anti-SARS-CoV-2 spike enzyme immunoassay [14, 15]. Testing was performed according to the manufacturer’s instructions at a certified biochemistry testing hospital laboratory. The lower limit of detection of the assay was 0.4 U/mL; according to the test instructions, any level > 0.8 U/mL was considered positive. For the purposes of this study, we regarded any subject at or below 0.8 as negative. According to the manufacturer’s specifications, neutralizing antibodies were assessed via the SARS-CoV-2 surrogate virus neutralization test assay (GenScript). Horseradish peroxidase-conjugated spike RBD was incubated with serum and then moved to angiotensin-converting enzyme 2-coated wells. Interactions of RBD and angiotensin-converting enzyme 2 were blocked if neutralizing antibodies [16]were present in the serum. The surrogate virus neutralization test measures the total quantity of neutralizing antibodies in the sera [17]. A positive result was defined based on a neutralizing antibody limit of ≥ 30% neutralization/inhibition. At this limit, the negative and positive percent agreement with the conventional plaque reduction neutralization test 50 and plaque reduction neutralization test 90 assays was approximately 100%. The sensitivity and specificity of these assays were 93.80% and 99.4%, respectively, according to the manufacturer’s instructions. According to the kit specifications, individuals with neutralization less than 30% were considered negative for neutralizing antibodies.

### Safety and adverse events

Our secondary endpoints were solicited specific local or systemic adverse events within 7 days after the receipt of each dose of the vaccine, and unsolicited adverse events within 30 days after the receipt of the second dose of the vaccine (Additional file 11: Appendix A).

The study team members contacted all participants within 1 week of the receipt of each dose by phone to collect any adverse events. The data were collected at each scheduled visit (Additional file 1: Appendix A) to assess episodes of acute allograft rejection, hospitalization, other adverse events, or COVID-19 infection during the entire duration of the study.

### Statistical analysis

The immunogenicity analysis was performed two weeks after the receipt of the first dose and 2 weeks after the receipt of the second dose for patients who received both vaccine doses and returned for follow-up. A safety analysis was performed for all patients, regardless of the number of doses administered. Demographic and safety analyses were performed using descriptive statistics. The primary outcome was vaccine immunogenicity assessed according to the anti-RBD titer two weeks after each dose of the vaccine, and will be further adjusted using propensity score analysis. A positive anti-RBD response was defined as > 0.8 U/mL. Univariate analyses were performed to determine factors impacting the development of a positive anti-RBD titer using the χ^2^ or Fisher’s exact test for categorical variables and we analyzed for changes in the lab parameters between screening and before the 2nd dose, and between screening and after the 2nd dose via t-tests. Statistical significance was defined as *p* < 0.05. All statistical analyses were performed using Stata version 17.0 (College Station, TX, USA).

The primary immunogenicity endpoint was considered the most important factor determining the necessary number of participants for this study. Furthermore, the endpoint was assumed to be binary for sample size calculations; that is, the recruited participant either did or did not achieve a sufficient antibody titer level 2 weeks after the second dose.

Multivariable logistic regression analyses were performed to simultaneously investigate the relationship between subgroups and the rate of immunogenicity. Similar analyses were performed to determine the efficacy outcomes (i.e., infection).

## Results

### Patient characteristics

Our cohort included 431 participants. Of these, 283 received the BNT-162b2 vaccine and 148 patients received the ChAdOx1 vaccine (230 kidney transplant recipients and 201 liver transplant recipients). The median age was 51.3 (± 16.2) years and 295 (68.4) were male. None reported a known history of COVID-19 prior to vaccination. All patient had stable graft function at the time of the vaccine. The baseline characteristics of the patients are described in Table 1. No significant differences in baseline characteristics were noted except for age (p > 0.00001) (Table 1).

**Table 1:** Baseline demographic and clinical characteristics of the population

Characteristic		Before propensity score matching			After propensity score matching		
	Total N = 431 (%)	Pfizer (n = 283)	AstraZeneca (n = 148)	p-value	Pfizer (n = 148)	AstraZeneca (n = 148)	p-value
Age (years)	51.3 ± 16.2	53.2 ± 16	47.7 ± 15.9	0.0008	46.9 ± 15.9	47.7 ± 15.9	0.675
Sex							
Male	295 (68.4)	197 (69.6)	98 (66.2)	0.504	102 (68.9)	98 (66.2)	0.619
BMI	28.2 ± 5.6	28.1 ± 5.5	28.2 ± 5.7	0.930	27.5 ± 5.9	28.2 ± 5.7	0.345
Hypertension	205 (47.5)	126 (44.5)	79 (53.3)	0.080	87 (58.7)	79 (53.3)	0.349
Diabetes	191 (44.3)	126 (44.5)	65 (43.9)	0.905	52 (35.1)	65 (43.9)	0.122
Type of Tx				0.000			0.898
Liver Kidney	201 (46.6) 230 (53.3)	158 (55.8) 125 (44.1)	43 (29) 105 (70.9)		44(29.7) 104 (70.2)	43 (29.05) 105 (70.95)	
Time since TX (years)	7.35 [0.13–33.4]	7.22 [0.13–33.4]	7.62 [0.5–22.7]	0.489	7.1 [0.13–33.4]	7.6 [0.5–22.7]	0.470
Tx < 1 year	9 (2)	8 (2.8)	1 (0.6)	0.138	3 (2.03)	1 (0.68)	0.314
Deaths	6 (1.3)	5 (1.7)	1 (0.6)	0.708	2 (1.35)	1 (0.68)	1.00
Prednisone	289 (67)	179 (63.2)	110 (74.3)	0.020	116 (78.3)	110 (74.3)	0.412
Tacrolimus	408 (94.6)	268 (94.7)	140 (94.5)	0.963	141 (95.2)	140 (94.9)	0.791
Mycophenolate	305 (70.7)	197 (69.6)	108 (72.9)	0.466	111 (75)	108 (72.9)	0.691
Triple regimen (TMP)^a^	235 (54.5)	146 (51.9)	89 (60.14)	0.091	94 (63.5)	89 (60.1)	0.550
Thymoglobulin^b^	133 (57.8)	76 (60.8)	57 (54.2)	0.31	64 (61.5)	57 (54.2)	0.288
Basiliximab	45 (10.4)	26 (9.1)	19 (12.8)	0.23	17 (11.4)	19 (12.8)	0.72

^a^TMP: tacrolimus, mycophenolate and prednisone

^b^Kidney Tx recipients

### Immunosuppression

The primary immunosuppressive regimen in the majority of the cohort composed of tacrolimus, mycophenolate and prednisone 235 (54.5%). With 408 (94.6%) of the patients were on tacrolimus as the cornerstone immunosuppressant. The immunosuppression intensity had the same impact on the vaccine response rate according to the neutralizing antibody (Table 1).

### Vaccine immunogenicity according to the neutralizing antibody

All patients were screened for COVID-19 before enrollment. Baseline laboratory test results and graft function were also assessed. There was no difference between patient’s laboratory parameters from baseline and two weeks following each dose of the vaccine (Table 2).

**Table 2:** A: Changes in patients laboratory value:

Overall					
	Screening	Before 2nd dose		After 2nd dose	
Parameter	Mean (SE)	Mean (SE)	P-value	Mean (SE)	P-value
HB	137.26 (0.96)	135.56 (1.56)	0.712	138.28 (1.93)	0.772
Platelet	242.04 (3.78)	238.71 (6.43)	0.349	227.92 (7.51)	0.90
INR	1.05 (0.01)	1.07 (0.02)	0.083	1.03 (0.01)	0.0425
Serum Creatinine	102.59 (2.79)	103.54 (4.57)	0.251	106.41 (7.12)	0.618
ALT	21.6 (0.68)	23.34 (1.13)	0.237	23.36 (3.3)	0.768
AST	19.3 (0.36)	21.04 (0.96)	0.611	18.87 (1.03)	0.889
ALK	98.56 (2.74)	109.92 (5.29)	0.722	94.62 (4.29)	0.817
GGT	57.27 (4.79)	74.17 (9.62)	0.918	62.03 (9.75)	0.436
Bilirubin total	10.17 (0.59)	10.39 (1.33)	0.734	9.09 (0.47)	0.083
Tacrolimus level	6.17 (0.16)	6.31 (0.31)	0.842	6.1 (0.36)	0.812
Pfizer					
HB	137.23 (1.2)	135.56 (1.82)	0.980	136.98 (2.22)	0.517
Platelet	235.24 (4.61)	236.95 (7.45)	0.709	224.96 (8.61)	0.69
INR	1.04 (0.01)	1.06 (0.02)	0.033	1.03 (0.01)	0.08
Serum creatinine	102.82 (3.79)	103.71 (6.03)	0.527	108.57 (8.34)	0.477
ALT	21.6 (0.83)	23.18 (1.34)	0.409	23.51 (3.91)	0.763
AST	19.49 (0.43)	21.13 (1.1)	0.541	18.89 (1.21)	0.844
ALK	100.59 (3.58)	113.85 (6.72)	0.727	92.95 (4.58)	0.778
GGT	62.7 (6.14)	75.36 (10.86)	0.972	56.22 (8.82)	0.138
Bilirubin total	10.71 (0.81)	11.04 (1.77)	0.647	9.33 (0.54)	0.047
Tacrolimus level	6.07 (0.19)	5.82 (0.32)	0.139	6.09 (0.38)	0.858
AstraZeneca					
HB	137.31 (1.62)	135.58 (3.08)	0.4106	143.73 (3.51)	0.629
Platelet	254.98 (6.46)	243.87 (12.84)	0.231	241.05 (14.49)	0.22
INR	1.1 (0.04)	1.12 (0.09)	0.455	1.03 (0.04)	0.1723
Serum creatinine	102.14 (3.69)	103.06 (4.23)	0.228	96 (10.02)	0.7632
ALT	21.59 (1.18)	23.84 (2.06)	0.365	22.7 (4.2)	0.986
AST	18.93 (0.64)	20.78 (1.96)	0.968	18.8 (1.55)	0.784
ALK	94.69 (4.13)	98.64 (6.69)	0.943	102.3 (11.72)	0.909
GGT	44.44 (6.8)	68.7 (20.7)	0.73	97.56 (43.91)	0.236
Bilirubin total	9.16 (0.75)	8.5 (0.65)	0.611	7.98 (0.85)	0.315
Tacrolimus level	6.35 (0.26)	7.63 (0.71)	0.032	6.12 (1.12)	0.8714

B: After propensity score matching					
Overall (n = 296)					
	Screening	Before 2nd dose		After 2nd dose	
Parameter	Mean (SE)	Mean (SE)	P-value	Mean (SE)	P-value
HB	137.19 (1.17)	136.39 (2.05)	0.750	139.9 (2.55)	0.311
Platelet	248.62 (4.67)	246.83 (9.2)	0.345	240.13 (10.59)	0.124
INR	1.07 (0.02)	1.05 (0.04)	0.868	1.03 (0.02)	0.055
Serum Creatinine	103.69 (2.94)	103.2 (3.72)	0.167	98.16 (4.26)	0.525
ALT	20.84 (0.81)	21.7 (1.21)	0.104	20.13 (1.83)	0.140
AST	18.63 (0.42)	19.7 (0.96)	0.877	18.16 (0.91)	0.115
ALK	96.57 (3.07)	105.28 (6.39)	0.280	100.69 (6.94)	0.420
GGT	51.92 (5.78)	78.15 (15.36)	0.993	86.65 (20.66)	0.6311
Bilirubin total	9.53 (0.52)	9.45 (0.56)	0.501	9.39 (0.66)	0.350
Tacrolimus level	6.35 (0.18)	6.67 (0.42)	0.286	6.11 (0.42)	0.9926
Pfizer					
HB	137.06 (1.68)	137.02 (2.76)	0.932	137.68 (3.47)	0.370
Platelet	242.18 (6.73)	249.12 (13.04)	0.778	239.64 (14.43)	0.296
INR	1.04 (0.01)	1.01 (0.02)	0.663	1.03 (0.02)	0.197
Serum Creatinine	105.23 (4.58)	103.3 (5.77)	0.401	99.21 (4.16)	0.33
ALT	20.09 (1.11)	20.12 (1.43)	0.162	18.82 (1.76)	0.057
AST	18.33 (0.54)	18.9 (0.85)	0.765	17.84 (1.14)	0.021
ALK	98.46 (4.56)	110.32 (10.02)	0.252	99.87 (8.72)	0.273
GGT	59.79 (9.43)	85.15 (22.14)	0.911	79.64 (20.34)	0.339
Bilirubin total	9.91 (0.73)	10.17 (0.85)	0.612	10.09 (0.89)	0.90
Tacrolimus level	6.36 (0.26)	5.91 (0.46)	0.389	6.11 (0.38)	0.888
AstraZeneca					
HB	137.31 (1.62)	135.58 (3.08)	0.4106	143.73 (3.51)	0.629
Platelet	254.98 (6.46)	243.87 (12.84)	0.231	241.05 (14.49)	0.22
INR	1.1 (0.04)	1.12 (0.09)	0.455	1.03 (0.04)	0.1723
Serum creatinine	102.14 (3.69)	103.06 (4.23)	0.228	96 (10.02)	0.7632
ALT	21.59 (1.18)	23.84 (2.06)	0.365	22.7 (4.2)	0.986
AST	18.93 (0.64)	20.78 (1.96)	0.968	18.8 (1.55)	0.784
ALK	94.69 (4.13)	98.64 (6.69)	0.943	102.3 (11.72)	0.909
GGT	44.44 (6.8)	68.7 (20.7)	0.73	97.56 (43.91)	0.236
Bilirubin total	9.16 (0.75)	8.5 (0.65)	0.611	7.98 (0.85)	0.315
Tacrolimus level	6.35 (0.26)	7.63 (0.71)	0.032	6.12 (1.12)	0.8714

### Factors associated with a lack of response to the vaccine

Factors previously reported to have affected seroresponse such as younger age, gender and time from transplantation were not clearly associated with response in our cohort. However, diabetes and triple immunosuppressive therapy appears to have significantly affected the response (Table 3).

**Table 3:** Anti-RBD levels: demographic factors (univariable analyses of factors associated with dose response)

	Before propensity score matching				After propensity score matching			
Characteristic	Response to dose-1 (%)	p-value	Response to dose-2 (%)	p-value	Response to dose-1 (%)	p-value	Response to dose-2 (%)	p-value
Male	59 (71.08)	0.198	78 (71.56)	0.295	32 (71.1)	0.365	46 (67.6)	0.759
Hypertension	38 (45.78)	0.194	53 (48.62)	0.252	29 (64.4)	0.430	38 (55.8)	0.419
Diabetes	35 (42.17)	0.898	49 (44.95)	0.040	12 (26.6)	0.108	25 (36.7)	0.023
Triple regimen (TMP)	27 (32.53)	0.000	52 (47.71)	0.000	21 (46.6)	0.000	41 (60.2)	0.003
Age^a^	1.02	0.018	0.979	0.079	0.99	0.985	0.96	0.028
Time since Tx^a^	0.99	0.740	1.007	0.831	0.99	0.907	0.96	0.377
HBV^b^	13 (24.53)	0.605	17 (32.08)	0.036	5 (33.3)	0.201	6 (31.5)	0.254

^a^Odds ratio

^b^In liver transplant patients only

A multivariable logistic regression was used including the same factors and demonstrated a pseudo R-square value of 0.23. Triple immunosuppressive therapy and age were identified as significant contributors for lack of response to the vaccine after the second dose with those receiving triple therapy having 92% reduced odds of a response and the per unit (year) increase in age associated with a 5% reduction in the odds of a response (Table 4).

**Table 4:** Multivariable logistic regression: factors associated with lack of response to the vaccine

Variable	Coefficient	OR (95% CI)	p-value
Female	− 0.618466	0.53 [0.22–1.30]	0.169
Hypertension	− 0.6821834	0.50 [0.18–1.40]	0.189
Diabetes	− 0.7022861	0.49 [0.21–1.19]	0.117
Triple regimen (TMP)	− 2.495359	0.08 [0.02–0.34]	0.000
Vaccine type: AstraZeneca	− 0.084671	0.91 [0.35–2.39]	0.862
Organ: Kidney	− 0.682618	0.50 [0.12–2.19]	0.362
Age	− 0.0488491	0.95 [0.92–0.98]	0.003
Time since Tx	0.0196044	1.01 [0.93.12]	0.683

### Anti-RBD levels by vaccine type

In our cohort, the response rate after the first vaccine dose appeared to be higher with Pfizer vaccine (P < 0.0001). However, this elevation did not persist until after the second dose (P = 0.863) (Table 5).

**Table 5:** Anti-RBD levels by vaccine type:

		Before propensity score matching			After propensity score matching		
Vaccine response	Total N = 431(%)	Pfizer (n = 283)	AstraZeneca (n = 148)	p-value	Pfizer (n = 148)	AstraZeneca (n = 148)	p-value
Post dose-1 Response after dose-1	33.20	41.61	17.98	0.000	31.8	17.98	0.031
Post dose-2 Response after dose-2	70.32	70.69	69.23	0.863	68.3	69.23	0.925
Kidney Tx Post dose-1 Response to dose-1	19.11	23.17	14.67	0.176			
Kidney Tx Post dose-2 Response to dose-2	60.87	59.38	64.29	0.657			
Liver Tx Post dose-1 Response to dose-1	56.99	60.76	35.71	0.081			
Liver Tx Post dose-2 Response to dose-2	84.13	84.62	81.82	0.818			

However, type of organ transplant significantly affected the response rate in our cohort (p = 0.002) (Table 6).

**Table 6:** Anti-RBD levels by type of Tx

	Kidney Tx: %	Liver Tx: %	p-value
Total			
Response to dose-1	19.11	56.99	0.000
Response to dose-2	60.87	84.13	0.002
Pfizer			
Response to dose-1	23.17	60.76	0.000
Response to dose-2	59.38	84.62	0.003
AstraZeneca			
Response to dose-1	14.67	35.71	0.060
Response to dose-2	64.29	81.82	0.286

### Change in spike antibody serology

The median antibody level before the second dose was 0.4 and after the second dose was 82.2. The median change in antibodies from before the second dose to after the second dose was 10.1

### Incidence of COVID-19

**Fig. 1:**
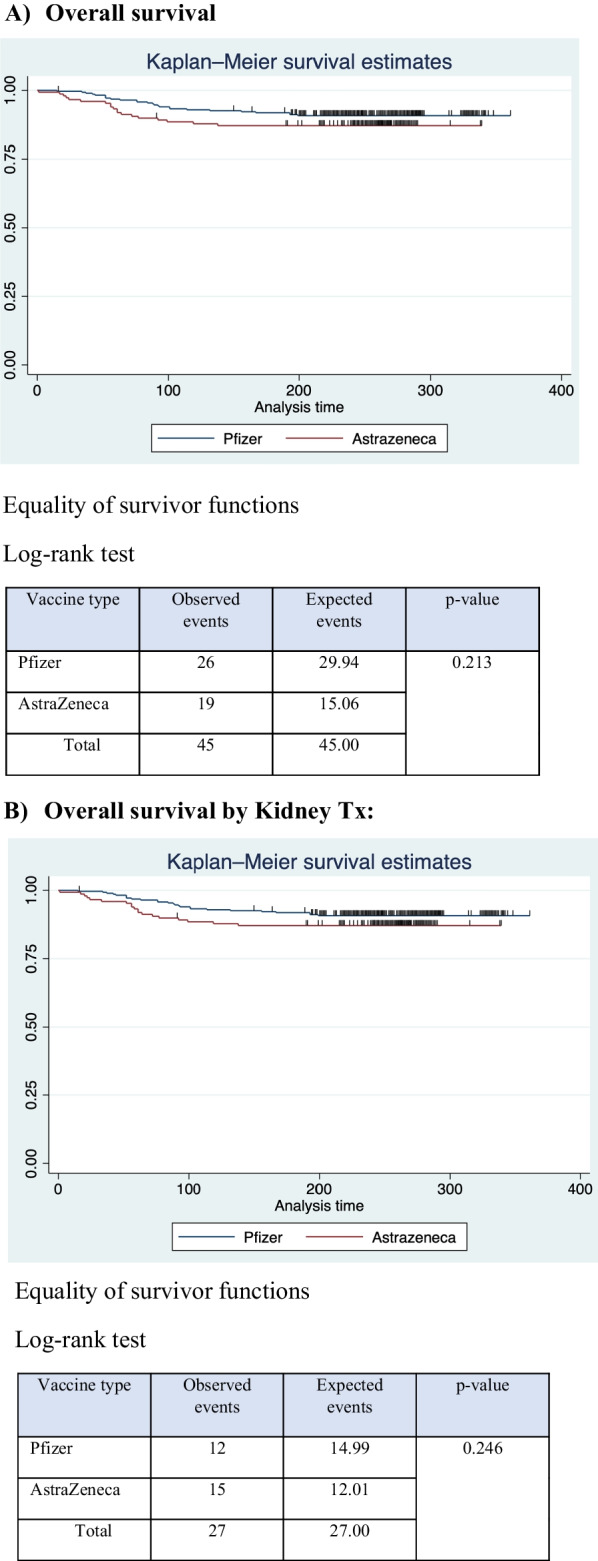

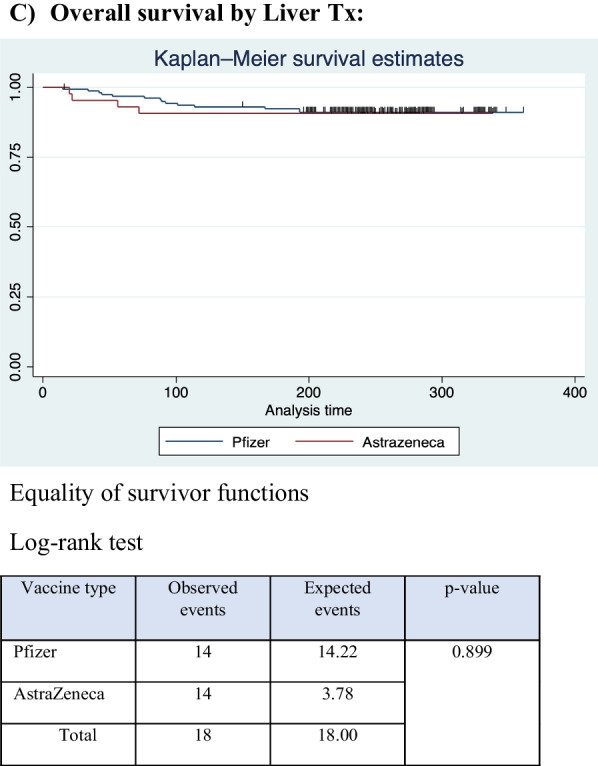
Time to infection outcome

A total of 45 cases of COVID-19 were confirmed by polymerase chain reaction in this cohort; these cases occurred in 19 of 148 participants who received the AstraZeneca vaccine and in 26 of the 283 participants who received the Pfizer-BioNTech vaccine. P = 0.213 (Fig. 1; Table 7).

**Table 7:** Anti-RBD levels by infection:

		Before propensity score matching			After propensity score matching		
	Total n (%)	Breakthrough n (%)	No breakthrough n (%)	p-value	Breakthrough n (%)	No breakthrough n (%)	p-value
Post dose-1 Response after dose-1	33.20	41.6	32.3	0.354	27.7	24.6	0.774
Post dose-2 Response after dose-2	70.32	90	68.97	0.159	100	66.6	0.088
Kidney Tx Post dose-1 Response to dose-1	19.11	25	18.4	0.527			
Kidney Tx Post dose-2 Response to dose-2	60.87	80	59.77	0.367			
Liver Tx Post dose-1 Response to dose-1	56.99	75	55.2	0.282			
Liver Tx Post dose-2 Response to dose-2	84.13	100	82.76	0.311			

### Vaccine safety and other outcomes

No evidence of graft dysfunction or rejection, or any other form of abnormality was observed in the entire cohort as evident by routine laboratory monitoring (Table 2). There were no significant changes in liver enzymes or liver function test results in the liver transplant population throughout the study period. There were no changes in serum creatinine levels in the kidney transplant population that necessitated any kidney allograft biopsy or further investigation. All side effects that occurred were grade 1 (mild) [18, 19], no medical intervention/therapy required. in this study were consistent with what’s been reported previously. Pain at injection site and fatigue occurred mainly with ChAdOx1 vaccine (Table 8).

**Table 8:** A: Adverse Drug Reactions

	Overall		Frequency		
	Freq	Percent	Pfizer	AstraZeneca	Signif
Following first vaccine dose (n = 431)					
Hypersensitivity	1	0.23	0	1	0.343
Bells palsy	0	–	0	0	
Gastrointestinal	2	0.46	0	2	0.117
Local pain at site	162	37.59	133	29	0.000
Headache/Fatigue	221	51.28	139	82	0.215
Neuromuscular skeletal	1	0.23	0	1	0.343
Dermatologic	0	–	0	0	
Miscellaneous	171	39.68	110	61	0.636
None	186	43.16	125	61	0.557
Following second dose (n = 410)					
Hypersensitivity	1	0.24	1	0	1.000
Bells palsy	0	–	0	0	
Gastrointestinal	1	0.24	1	0	1.000
Local pain at site	72	17.56	62	10	0.000
Headache/Fatigue	61	14.88	51	10	0.002
Dermatologic	0	-	0	0	
Miscellaneous	65	15.85	54	11	0.001
None	315	76.83	191	124	0.000

B: Adverse drug reactions after propensity score matching					
	Overall		Frequency		
	Freq	Percent	Pfizer	AstraZeneca	Signif
Following first vaccine dose (n = 269)					
Hypersensitivity	1	0.34	0	1	1.00
Bells palsy	0	–	0	0	
Gastrointestinal	2	0.68	0	2	0.498
Local pain at site	79	26.6	50	29	0.006
Headache/Fatigue	131	44.26	49	82	0.00
Neuromuscular skeletal	1	0.34	0	1	1.00
Dermatologic	0	–	0	0	
Miscellaneous	96	32.4	35	61	0.001
None	144	48.6	65	87	0.01
Following second dose (n = 277)					
Hypersensitivity	0	–	0	0	
Bell’s palsy	0	–	0	0	
Gastrointestinal	1	0.36	1	0	0.495
Local pain at site	31	11.19	21	10	0.031
Headache/Fatigue	28	10.11	18	10	0.098
Dermatologic	0	-	0	0	
Miscellaneous	32	11.5	21	11	0.052
None	230	83.03	106	124	0.013

## Discussion

A key strength of our study is the head-to-head evaluation and comparison of the serologic response to the BNT162b2 mRNA and ChAdOx1 vaccines against COVID-19 in a large transplant cohort in a prospective fashion. Our key finding is that both vaccine platforms provide comparable anti-Spike levels against COVID19 infection, even after adjusting with propensity score matching. On the other hand, previously reported factors that may have an impact on vaccine responsiveness were not evident in our cohort [20, 21].

It is not yet clear whether these antibody responses will be adequate to protect transplant recipients from symptomatic COVID-19. Associations between neutralizing activity and clinical protection were not evaluable in this study due to the small number of breakthrough infection in the cohort.

Another point of originality of our study is that, we showed that both vaccine platforms were safe, and have comparable side effect profile. We have also noticed that BNT-162b2 vaccine may produce higher titers numerically, especially after first dose, this effect did not persist after the second dose. A previous study examined the outcomes of the Ad26.COV2. S vaccine compared to those of the mRNA vaccine; only 2 of 12 participants who received a single dose of the Ad26.COV2. S vaccine had a detectable anti-RBD antibody response, which was significantly fewer than the observed number of recipients with a detectable anti-RBD antibody response who received the mRNA vaccine series. Additionally, the titers achieved by the Ad26.COV2. S groups were significantly lower than those achieved by the mRNA group [22]. One potential explanation of the lower titer level after the first dose in the ChAdOx1 arm is that, in clinical trials, antibody titers usually peak at 21 days after receipt of the first dose [23], our study protocol measures the titers two week after each dose of the vaccine.

During SARS-CoV-2 mRNA and virus vector vaccine studies involving the general population, seroconversion was observed in almost all patients [3, 4, 6–8, 15, 24]. However, as expected, the response rate was lower in our cohort than it was in the general population; this finding is consistent with the available data in the field [25–28]. Considering only the humoral response, spike-specific antibodies developed in only 29.9% of patients in our population, which is a bit lower than general population, and those with other immunocompromising conditions [29]. However, studies have reported a 37.5% antibody response rate after the second dose of the BNT162b2 vaccine. Boyarsky et al. reported a higher seroconversion rate of 54% for patients who received either the mRNA-1273 vaccine (Moderna^®^) or the BNT162b2 vaccine (Pfizer), both of which are mRNA vaccines [30]. Although no consensus on what threshold should be considered as protective immunity. In general, antibody levels were well below what has been reported in immunocompetent subjects.

It has been reported that the immune response to the vaccines was also impacted by the immunosuppressive protocol used [31, 32]. Some studies have addressed that anti-metabolite use (mycophenolate and azathioprine) are linked to poorer humoral responses to COVID-19 vaccines after SOT [33, 34]. Yet, the impact was consistent across vaccination platforms in our cohort. Moreover, we found that the odds of seropositivity among SOT patients receiving triple immunosuppressive regimen was lower compared to those receiving only 1 drug, irrespective of the pharmacological class. This implicates that the net state of immunosuppression, is the main predictor of poor humoral responses after SOT rather than a particular medication. We also found that seropositivity in kidney transplant recipients was lower than that of liver transplant recipients, which could also be explained by the intensity of immunosuppressive regimen used across organs.

It has also been observed that, in SOT recipients, the odds of seropositivity in patients who were vaccinated within 1 year after transplantation was lower than those who received the vaccines after the 1st year of transplantation [21]. This effect was not evident in our population, and was consistent across vaccine platforms.

The safety of both vaccine platforms especially vector vaccines in solid organ transplant recipients was another point of concern amongst healthcare providers. Our findings match those reported in the original trials of the BNT162b2 vaccines. Pain at the injection site, fatigue, and headache were the most common symptoms experienced by healthy adults and those with stable, chronic medical conditions [31, 32]. None of the subjects in our large cohort experienced serious adverse events such as thrombocytopenia nor severe hypersensitivity reaction similar to what have been published [32, 35–38] Those findings shall eliminate hesitancy or preference of a particular vaccine platform over the other.

However, the concern remains whether the antibody titers correlate with the clinically meaningful protection. Therefore, the clinicians should inform the patients that the immune response following vaccination may not provide a full protection against COVID19 infection.

To the best of our knowledge, this is the first study that directly compared the efficacy of different vaccine platforms in solid organ transplant recipients. Our results suggest that solid organ transplant recipients should not be limited to COVID-19 vaccinations with mRNA platforms despite of the observed of the suppressed efficacy of viral vector vaccines, and that their antibody titers should be routinely checked to assess the response. At this point, the focus should continue to be vaccinating the family members and caregivers of solid organ transplant recipients as part of a cocooning strategy, which is a well-known method of protection when the target population cannot be vaccinated or is at risk for having a low response rate.

Limitations of this study include, lack of an immunocompetent control group, and lack of exploration of memory B-cell or T-cell responses. We also did not evaluate neutralizing antibody titers against the Delta or Omicron SARS-CoV-2 variants. Given that those variants were not reported at the time of the conduct of the study. Moreover, vaccine efficacy against these two variants is likely reduced [39–44].

## Supplementary Information

**Additional file 1: Supplementary Appendix A:** Schedule of Assessment.

## Data Availability

All data generated or analysed during this study are included in this published article.

## Abbreviations

ANC: Absolute neutrophil count
AE: Adverse event
ALT: Alanine aminotransferase
ANOVA: Analysis of variance
AST: Aspartate aminotransferase
AZA: Azathioprine
β − ηΧΓ: Beta-human chorionic gonadotropin
BCAR: Biopsy-proven acute rejection
BP: Blood pressure
BMI: Body mass index
CNI: Calcineurin inhibitor
CRF: Case report form
CI: Confidence interval
COVID-19: Coronavirus disease 2019
CIOMS: Council for International Organizations of Medical Sciences
CSA: Cyclosporine
CMV: Cytomegalovirus
DNA: Deoxyribonucleic acid
ECG: Electrocardiogram
eCRF: Electronic case report form
EBV: Epstein-Barr virus
eGFR: Estimated glomerular filtration rate
EMA: European Medicines Agency
FSH: Follicle-stimulating hormone
GCP: Good clinical practice
HBc Ab: Hepatitis B core antibody
HBV: Hepatitis B virus
HIV: Human immunodeficiency virus
HIV: Human immunodeficiency virus
ICU: Intensive care unit
