# Supplementary material for: Comparison of the safety and immunogenicity of the BNT-162b2 vaccine and the ChAdOx1 vaccine for solid organ transplant recipients: a prospective study
Source: BMC Infect Dis. 2022 Oct 13;22:786. doi: 10.1186/s12879-022-07764-x (PMC9559153; doi:10.1186/s12879-022-07764-x)
Supplement: Supplementary file 1 — Additional file 1: Supplementary Appendix A: Schedule of Assessment. [file 12879_2022_7764_MOESM1_ESM.docx]

## **Supplementary Appendix A: Schedule of Assessment**

| SCHEDULE OF EFFICACY ASSESSMENTS | | | | | | | | | | |
| --- | --- | --- | --- | --- | --- | --- | --- | --- | --- | --- |
| Procedure/timeline | **Screening Visit** | **FIRST DOSE** | **Week 2** | **SECOND DOSE** | **Week 2** | **Month 3** | **Month 6** | **Month 9** | **Month 12** |  |
| Informed consent | **X** |  |  |  |  |  |  |  |  |  |
| Medical history and demographic data | **X** |  |  |  |  |  |  |  |  |  |
| Review inclusion & exclusion criteria | **X** |  |  |  |  |  |  |  |  |  |
| Laboratory | **X** |  |  |  | **X** | **X** | **X** | **X** | **X** |  |
| Serology (AB) | **X** |  | **X** |  | **X** | **X** | **X** | **X** | **X** |  |
| Donor Specific Antibodies (DSAs) | **X** |  |  |  |  |  |  |  |  |  |
| Biopsy & Treatment of rejection | **When clinically indicated at the discretion of treating physician** | | | | | | | | | |
| COVID-19 PCR |  | | | | **When clinically indicated at the discretion of treating physician** | | | | | |
